# Emergency preparedness and disaster management for primary health care in Africa from the perspective of climate hazards and extreme weather events

**DOI:** 10.4102/phcfm.v18i1.5449

**Published:** 2026-05-20

**Authors:** Elzarie Theron, Wayne P. Smith

**Affiliations:** 1 Division of Emergency Medicine, Faculty of Health Science, University of Cape Town, Cape Town, South Africa; 2 Department of Health and Wellness, Western Cape Government, Cape Town, South Africa

**Keywords:** climate hazards, extreme weather events, primary health care, disaster preparedness, disaster risk management

## Abstract

Climate-related hazards are impacting community health and healthcare provision across the African continent, resulting in increased vulnerability and reduced capacity to withstand further impacts. While it is important to recognise that not all climate-related hazards result in disaster, this is often the case in populations that are already vulnerable, creating a vicious cycle that further increases risk. However, with appropriate multisectoral collaboration, planning and preparedness, community resilience can be strengthened, thereby reducing the likelihood and severity of disasters. Situated at the interface between health systems and communities, primary care providers are uniquely placed to identify climate-related vulnerabilities and emerging risks. Sharing these insights through disaster risk management platforms can inform planning processes and strengthen preparedness and resilience. With this purpose in mind, this article outlines key concepts and approaches in disaster risk management, supported by a real-life case study that illustrates lessons learned and their implications for primary health care, with the aim of better equipping providers to participate in and support resilience-building efforts.

## Introduction

Climate change is causing an increase in the frequency and severity of extreme weather events.^1^ These events have several direct impacts on health and healthcare facilities^2,3,4,5,6^ and have been linked to shifts in patterns of injury, disease and mortality.^7^ Direct health impacts from extreme weather events, such as floods, droughts and heatwaves, often cause injuries or death and other more indirect health impacts, such as cardiovascular morbidity.^1,8^ These impacts tend to disproportionately affect vulnerable populations.^9^

It is important to recognise that not all climate-related hazards or extreme weather events necessarily lead to disasters; with adequate planning, preparedness and management, many disasters can be prevented or their impacts significantly reduced. Appropriate planning for the effective management of disaster risk helps protect the communities we serve. Evidence shows that with the necessary disaster risk reduction (DRR) planning in place, not only are lives saved, but livelihoods are safeguarded, enabling communities to become more resilient to these events.^10,11^

In this article, we outline key concepts and approaches to improve primary care providers’ (PCPs’) understanding of disaster risk and how vulnerability develops within a specific community. We also discuss how this understanding can inform multisectoral and multilevel DRR efforts and the importance of collaboration to strengthen community resilience to climate-related hazards. A case study from KwaZulu-Natal, South Africa, is presented to highlight lessons learned from a previous disaster event. Finally, we outline the role of primary health care (PHC) in supporting preparedness and response to climate-related disasters.

## Disaster risk

Disasters occur at the interface between an extreme physical event and a vulnerable human population^12,13,14^ when a disruption goes beyond the capacity of the affected community or society to cope using its available resources.^15^ In other words, disasters occur when a group of vulnerable people are exposed to a hazard and suffer severe disruption and/or damage to their livelihood system in a way that they are unable to recover from without external aid.^14^ The occurrence of a disaster, therefore, lies in the susceptibility of the affected human population to a hazardous event.

Traditionally, disasters were approached using the disaster management cycle, which has a disaster-oriented focus.^16^ Efforts revolved around response and relief following a disaster.^17^ A shift in perspective occurred during the 1970s when an increase in disasters led to greater economic and human losses and impaired human development, catalysing a paradigm shift from disaster management to disaster risk management (DRM).^17,18^ People’s vulnerability to hazards became a focal point in the discussion about DRM.^16,19^

The United Nations Office for Disaster Risk Reduction (UNISDR) defines vulnerability as ‘the characteristics and circumstances of a community, system or asset that puts it at risk to the damaging effects of a hazard’.^15^ The vulnerability of a community is often rooted in social, economic and political processes, and contexts that exist independently of the hazardous event itself.^14^ This includes the distribution of income, assets and access to resources – such as healthcare, information, welfare and social protection – among different groups within a society.^14^ For example, adverse economic and political conditions may compel people to live in areas frequently exposed to climate-related hazards, thereby increasing their vulnerability to events such as floods or wildfires and, consequently, the overall risk of disaster.

On the other hand, capacity refers to the characteristics and strengths present in a community that help to reduce vulnerability.^14,15^ Examples include local knowledge about weather patterns, early warning systems for climate shocks and trained healthcare personnel to respond to climate-related health impacts.

Primary care providers are embedded within communities and are therefore well-positioned to identify the conditions that shape vulnerability. An understanding of the factors that produce vulnerability and the capacities that can be mobilised to reduce it is an important step in the development of DRR plans. It is therefore essential that PCPs understand the progression of vulnerability and how it manifests within the communities they serve. Each vulnerability assessment must be conducted with a specific community in mind, recognising that the factors contributing to vulnerability in one setting may differ substantially from those in another and may vary depending on the hazard(s) affecting that area.

In the next section, we will discuss a framework that explains how disasters occur when climate-related hazards interact with vulnerable conditions in a community. For PCPs, it provides a structured way of identifying the key areas to examine in order to understand vulnerability to a specific hazard within a particular community or health facility.

## Understanding disaster risk manifestation through the pressure and release model

The pressure and release (PAR) model is a conceptual framework used in DRM that explains how the progression of vulnerability, when combined with a hazard, produces disaster risk.^14^ According to Wisner et al., in assessing disaster risk, the progression of vulnerability – which includes social, economic and political causes and pressures – demands at least the same level of attention as the analysis of natural hazards.^14^

From the PAR perspective, disaster risk is a product of the interaction between a hazard and vulnerability.^20^ The central assumption of the PAR model is that disaster risk emerges from the interplay of two opposing forces: root causes, dynamic pressures and unsafe conditions that generate vulnerability on the one hand, and a hazardous event on the other.^14^ The notion of ‘release’ refers to the reduction in disaster risk, which necessitates the reduction in vulnerability.^14^

The PAR model comprises five components. The first three represent the progression of vulnerability – namely, root causes, dynamic pressures and unsafe conditions. The remaining two capture the hazard and the resulting disaster, which arises from the interaction between the first three components and a specific hazard.^14^

Root causes are the most remote of the three components and can manifest in several ways. Spatially, they may be linked to economic or political power (e.g., unequal distribution of resources that may result in rural PHC facilities with fewer staff or insufficient infrastructure). Temporally, they may be rooted in historical contexts that continue to shape present-day systems (e.g., historical patterns of development or governance that result in unequal access to primary care services). Socio-culturally, they may relate to cultural assumptions and ideologies so deeply ingrained in society that they are often overlooked (e.g., social norms or beliefs that influence how and when individuals seek care).^14^ Root causes emphasise the role of power distribution within a society and its implications for access to resources, and the impact of hegemonic ideologies, particularly around political and economic systems.^14,20^

The next component in the progression of vulnerability is dynamic pressure. These are external processes that translate root causes into specific unsafe conditions.^14^ Dynamic pressures must be examined in relation to the hazards impacting a specific community and may include rapid population growth, rural–urban migration, ongoing (as opposed to historical) conflict and epidemic disease.^14^ In a primary care context, these pressures may look like an increased demand for services, strain on limited workforce capacity and disruptions to service delivery or in maintaining essential supplies or infrastructure.

The third component in the progression of vulnerability is unsafe conditions, which may manifest in several ways. In the physical environment, unsafe conditions may relate to hazardous locations and inadequate infrastructure (e.g., a PHC facility located in a flood-prone area or lacking reliable electricity or water supply). In the local economy, they may be linked to low income and rising housing costs (e.g., economic constraints that limit patients’ ability to access healthcare services). In social relations, unsafe conditions may arise where institutions and social networks are weak (e.g., limited coordination between PHC facilities, community structures and disaster management agencies). Within public policies and institutions, unsafe conditions may occur where disaster preparedness plans are insufficient (e.g., the absence of DRR or response plans within PHC facilities).^14^ Unsafe conditions, therefore, represent the specific ways in which the vulnerability becomes visible in relation to a particular hazard.^14^

**FIGURE 1:**
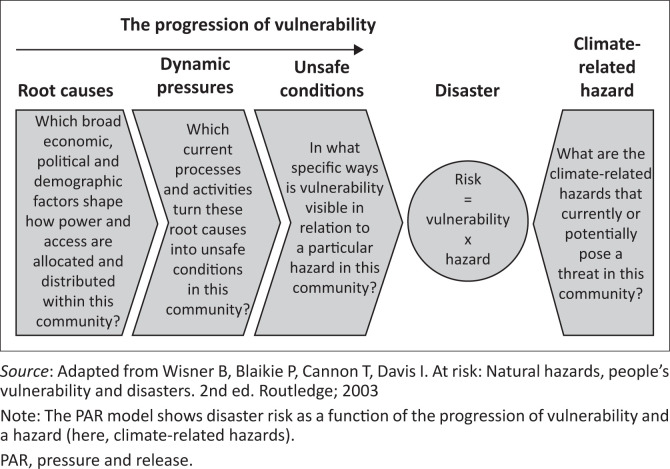
The pressure and release model.

Figure 1^14^ presents a series of core questions corresponding to each PAR component. These questions aim to guide PCPs in the development of a context-specific understanding of the progression of vulnerability in a specific community or PHC facility, which, through the interaction with specific climate-related hazards, increases the risk of a disaster in that context.

The PAR model provides a framework for identifying how vulnerability to a specific hazard develops within a community, enabling PCPs to understand risk beyond its visible symptoms. Building on this understanding, the following section outlines how disaster risks can be assessed and profiled within a community. While these processes usually involve multisectoral collaboration, the insights generated through vulnerability analysis at the PHC-level can contribute valuable contextual information, forming the basis for the development of appropriate DRR plans and measures.

## Disaster risk profiling and reduction planning

A disaster risk assessment (DRA) informs DRM by identifying the key disaster risks within a community through an assessment of its vulnerabilities, hazards and capacities. These assessments are typically undertaken through multisectoral collaboration and coordinated at the municipal or district level, with sectors such as PHC contributing relevant contextual information.

There are various methods available for collecting information about risks, ranging from reviews of historical data and hazard mapping^21^ to community-based disaster risk assessment (CBDRA) techniques, such as interviews with community members and transect walks.^22^ For more participatory risk assessment methods specifically in informal settlements, see Holloway et al.^23^ The findings from the DRA can then be used to develop a DRR plan aimed at strengthening resilience and reducing the risks associated with specific hazards.

While a disaster risk profile provides an overview of the key disaster risks within a specific community, a DRR plan uses this information to outline practical steps to increase preparedness for a hazardous event. A DRR plan often provides an overview of the hazards identified in the DRA (e.g., wildfire), along with their associated risks (e.g., air pollution), recommended DRR measures (e.g., developing guidelines for managing patients with asthma), assigned roles and responsibilities and required resources.

Finally, the UNISDR emphasises that monitoring and evaluation (M&E) is a critical process for ensuring the effectiveness of DRR plans and programmes.^24^ Monitoring and evaluation enables measurement and assessment of the performance of DRR plans against established goals or outcomes, ultimately leading to improved results. This process also facilitates the early identification of issues or challenges, allowing for timely adjustments to DRR plans to enhance its outputs and outcomes. By holding stakeholders accountable for their roles and responsibilities, M&E promotes the efficient use of available resources and supports evidence-informed decision-making.^24^ Another important component of the M&E process is a focus on *lessons learned*. By capturing key insights, successes and challenges that emerge during the implementation of the DRR plan the aim is to improve future DRR efforts.

In the following section, we present a real-life case study to illustrate how such lessons learned can emerge from disaster events and how they may inform DRR planning and collaboration within PHC contexts.

## Lessons learned from the KwaZulu-Natal floods: Implications for primary health centre emergency preparedness and disaster management

The KwaZulu-Natal floods of April 2022 serve as a stark illustration of climate-related vulnerability. Within a 24-h period between 11 April 2022 and 12 April 2022, approximately 300 mm of rainfall was recorded, leading to 544 fatalities and the displacement of around 42 000 people.^25^ This catastrophic event underscores both the human cost of climate extremes and the limitations of current disaster preparedness systems.

Following this event, the Presidential Climate Commission (PCC), an independent multistakeholder body established to guide South Africa’s climate response, was mandated to conduct a comprehensive review to strengthen national resilience and improve future response capacity.^26^ In South Africa, the *Disaster Risk Management Act* (*Act 57 of 2002*) provides a legislative framework intended to ensure coordination and interoperability among agencies responsible for disaster preparedness and response.^27^ However, the PCC report demonstrates that, despite this framework, challenges remain in ensuring effective implementation and intersectoral collaboration during large-scale disasters.

The PCC report emphasised that adaptive governance, continuous capacity building and improved knowledge-sharing mechanisms are central to building climate-resilient institutions.^26^ These lessons learned underscore the importance of collaboration across sectors, including PHC and the public, which collectively can strengthen resilience to future events.

Experiences from the Western Cape Department of Health and Wellness have demonstrated the benefits of multi-agency collaboration in enhancing climate and disaster resilience.^28^ Quintana et al. further highlight the importance of integrated communication and coordinated planning across government sectors.^29^ For example, early warnings issued by national meteorological services enable proactive mobilisation and preparedness for potential hazards, including floods and heatwaves. Collaboration also improves data integration for risk mapping. By combining meteorological data – such as rainfall, river levels and soil saturation – with demographic and infrastructural information, authorities can identify high-risk zones more accurately. These overlays guide evidence-based planning, allowing decision-makers to prioritise vulnerable areas, including informal settlements, and allocate resources effectively for response and recovery. In many contexts, these processes are facilitated by disaster management centres or similar coordinating bodies that convene stakeholders across sectors to ensure coordinated and timely responses. Such arrangements demonstrate the effectiveness of institutionalised collaboration in reducing the lag between early warning and community response.

Public engagement also remains a critical pillar of effective disaster risk management.^30^ For example, early warning information issued by national meteorological services must be translated into clear, trustworthy and actionable communication for communities at greatest risk, such as residents of informal settlements located along flood-prone riverbanks.^31^ Targeted outreach through diverse media platforms (e.g., radio, print materials, community forums and social media) can enhance early preparedness and promote protective behaviours. Such community-based interventions can not only reduce mortality and displacement during extreme weather events but also strengthen public trust in institutional response mechanisms.

As the first point of contact with communities, PHC not only plays a pivotal role in the surveillance and early identification of climate-related disaster risks, but also in communicating this information to key stakeholders. Harnessing these insights through meaningful participation in disaster risk management forums can support the integration of community-level knowledge into existing frameworks, thereby strengthening resilience.

**FIGURE 2:**
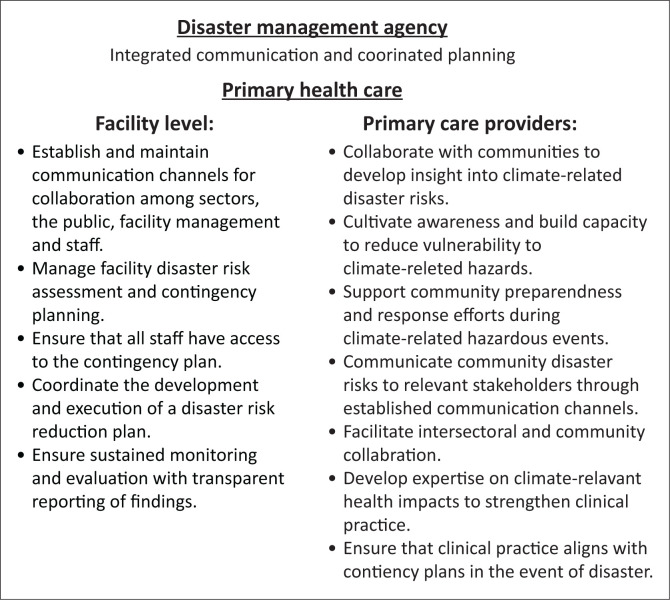
Primary health care contributions to climate-related disaster preparedness and response.

Figure 2 outlines key areas in which PCPs can contribute to climate-related disaster preparedness and response at both facility and community levels, informed by the lessons discussed in the case study.

## Conclusion

With health systems across the African continent increasingly vulnerable to the impacts of climate change, this article aimed to provide information for PCPs to enhance emergency preparedness and DRM in the context of climate-related hazards. Appropriate emergency preparedness and DRM can save lives, protect livelihoods and strengthen the resilience of communities and health systems.

This article, therefore, provides an overview of key concepts and approaches related to DRM to assist PCPs in strengthening their understanding of vulnerability and risk in relation to climate-related hazards. As primary providers of health in the community, PCPs are well-positioned to observe and identify climate-related disaster risks as they emerge and to relay this information to key stakeholders involved in the DRM process.

To support this understanding, we discuss the PAR model and provide guidance to assist PCPs in identifying the root causes, dynamic pressures and unsafe conditions that increase communities’ vulnerability to climate-related hazards.

This discussion is situated within the broader context of DRM, illustrating where PCPs contribute within the multisectoral and multilevel processes that characterise DRM. We provide a brief overview of community-based methods used in DRA and how these processes inform DRR planning. We also discuss the importance of M&E and lessons learned as mechanisms to strengthen future preparedness and response efforts.

A real-life case study is presented to illustrate lessons learned from a disaster event, which underline the importance of adaptive governance, ongoing capacity development and improved collaboration across sectors. Finally, we demonstrate how these insights can inform PHC preparedness and response at both facility and community levels.

In conclusion, the increasing frequency and severity of climate-related hazards highlight the urgency of reinforcing disaster management frameworks through multisectoral collaboration, knowledge exchange and community engagement. As the adage aptly reminds us, ‘disaster management is everyone’s concern’. Sustained collaboration, informed governance and resilient communities remain the foundation of an adaptive, climate-resilient Africa.
